# Does Treatment Order Matter? Investigating the Ability of Bacteriophage to Augment Antibiotic Activity against *Staphylococcus aureus* Biofilms

**DOI:** 10.3389/fmicb.2018.00127

**Published:** 2018-02-05

**Authors:** Dilini Kumaran, Mariam Taha, QiLong Yi, Sandra Ramirez-Arcos, Jean-Simon Diallo, Alberto Carli, Hesham Abdelbary

**Affiliations:** ^1^Center for Innovative Cancer Therapeutics, Ottawa Hospital Research Institute, Ottawa, ON, Canada; ^2^Centre for Innovation, Canadian Blood Services, Ottawa, ON, Canada; ^3^Division of Orthopedic Surgery, The Ottawa Hospital, Ottawa, ON, Canada

**Keywords:** bacteriophage, *Staphylococcus aureus*, biofilm, antibiotics, synergy

## Abstract

The inability to effectively treat biofilm-related infections is a major clinical challenge. This has been attributed to the heightened antibiotic tolerance conferred to bacterial cells embedded within biofilms. Lytic bacteriophages (phages) have evolved to effectively infect and eradicate biofilm-associated cells. The current study was designed to investigate the ability of phage treatment to enhance the activity of antibiotics against biofilm-forming *Staphylococcus aureus*. The biofilm positive *S. aureus* strain ATCC 35556, the lytic *S. aureus* phage SATA-8505, and five antibiotics (cefazolin, vancomycin, dicloxacillin, tetracycline, and linezolid), used to treat *S. aureus* infections, were tested in this study. The ability of the SATA-8505 phage to augment the effect of these antibiotics against biofilm-associated *S. aureus* cells was assessed by exposing them to one of the five following treatment strategies: (i) antibiotics alone, (ii) phage alone, (iii) a combination of the two treatments simultaneously, (iv) staggered exposure to the phage followed by antibiotics, and (v) staggered exposure to antibiotics followed by exposure to phage. The effect of each treatment strategy on biofilm cells was assessed by enumerating viable bacterial cells. The results demonstrate that the treatment of biofilms with either SATA-8505, antibiotics, or both simultaneously resulted in minimal reduction of viable biofilm-associated cells. However, a significant reduction [up to 3 log colony forming unit (CFU)/mL] was observed when the phage treatment preceded antibiotics. This effect was most pronounced with vancomycin and cefazolin which exhibited synergistic interactions with SATA-8505, particularly at lower antibiotic concentrations. This *in vitro* study provides proof of principle for the ability of phages to augment the activity of antibiotics against *S. aureus* biofilms. Our results also demonstrate that therapeutic outcomes can be influenced by the sequence in which these therapeutic agents are administered, and the nature of their interactions. Further investigation into the interactions between lytic phages and antibiotics against various biofilm-forming organisms is important to direct future clinical translation of efficacious antibiotic–phage combination therapeutic strategies.

## Introduction

The majority of human bacterial infections are thought to involve biofilm-associated bacterial pathogens (Römling and Balsalobre, 2012). Broadly defined, biofilms are communities of microbial cells adhered to biotic or abiotic surfaces encased in a self-produced matrix (Costerton et al., 1999). The ability to form biofilms has been shown to provide associated bacterial cells with heightened tolerance to antibiotics when compared to their planktonic counterparts. Biofilms account for the observed recalcitrance of biofilm-associated chronic bacterial infections (Mah and O’Toole, 2001). The heightened resistance displayed by biofilms is thought to be multifaceted, with the matrix serving as the first line of defense. The physical and biochemical properties of the matrix have been reported to impede the diffusion of antimicrobial agents into the biofilm which leads to suboptimal concentrations of these agents within the biofilm thereby reducing their efficacy (Gordon et al., 1988). Additionally, mature biofilms display physiochemical stratification caused by the varying availability of nutrients and waste products within the biofilm. As a result, cells found in the deeper layers of biofilms are generally less metabolically active than those found in the periphery, and are consequently less susceptible to antimicrobials that rely on active replication for their activity (Fauvart et al., 2011). The differential expression of genes within biofilms has also been shown to contribute to heightened antibacterial tolerance. This was observed in *Escherichia coli* and *Pseudomonas aeruginosa* biofilms, where efflux pumps and periplasmic glucans were upregulated, respectively (Mah et al., 2003; Lynch et al., 2007). Finally, the presence of a subset of isogenic cells called persister cells and naturally occurring antibiotic-resistant cells play a key role in the persistence of biofilms following antibiotic treatment. Persister cells become metabolically dormant and exhibit tolerance in the presence of antimicrobials; however, they are able revert to an active metabolic state in its absence (Lebeaux et al., 2014). These factors together with the ever-mounting threat of antibiotic resistance have made the search for alternative treatments of biofilm-related infections a high priority in several clinical disciplines including orthopedic surgery and cardiac surgery (Archer et al., 2011; Tande and Patel, 2014).

Bacteriophages (phages) are viruses that are highly specific to their bacterial hosts. They were discovered in the early 1900s (Salmond and Fineran, 2015) and were quickly shown to be effective in treating bacterial infections (Schultz, 1929; MacNeal and Frisbee, 1936). However, with the introduction of antibiotics, the appeal of phage therapy rapidly diminished. Due to the emergence of multi-drug-resistant bacterial pathogens in recent years, there has been renewed interest in phage therapy as an alternative antimicrobial strategy (Doss et al., 2017). Phages co-evolve with bacteria in nature; consequently, phages have developed mechanisms to overcome the obstacles posed by the biofilm state. Some of these mechanisms include exploiting water channels within the biofilm to penetrate into the deeper layers of the biofilm (Doolittle et al., 1996), or the expression of depolymerases that can disrupt the extracellular matrix allowing phage to penetrate and spread within the biofilm (Parasion et al., 2014). Biofilms also provide an excellent niche for phage replication since bacteria are found at high densities. Therefore, phages can self-amplify and reach high concentrations at the site of infection with a low initial dose (Burrowes et al., 2011). Phages have also been shown to infect antibiotic-resistant bacterial cells, since the evolved resistance mechanisms against antibiotics do not affect phage infection. As a result, the utilization of phage to treat infections caused by these resistant bacterial cells can help eliminate the selection of these cells and consequently minimizes persistence (Loc-Carrillo and Abedon, 2011). Additionally, Pearl et al. (2008) demonstrated that though phages require metabolically active hosts to replicate, they can infect persister host cells where they remain dormant. However, the phage lytic cycle is activated upon reversion to an active metabolic state, thereby abrogating the risk of reseeding.

A notable example of a human pathogen that is able to cause biofilm-related chronic infections is the commensal opportunistic bacterium *Staphylococcus aureus*. This bacterium is the leading cause of biofilm-related infections associated with implanted medical devices, such as heart valves, catheters, and prosthetic joints (Archer et al., 2011; Tande and Patel, 2014). In an effort to combat these recalcitrant infections, studies have investigated the potential of using matrix dispersal agents in conjunction with antibiotics (Lauderdale et al., 2010; Reffuveille et al., 2014). However, several shortcomings of this approach include the presentation of suboptimal levels of antibiotics within the biofilm, which lead to either acute infections or inadvertent upregulation of biofilm-forming genes (Lister and Horswill, 2014). Encouragingly, studies have demonstrated that a *S. aureus*-specific phage can successfully treat *S. aureus* infections when used in conjunction with antibiotics (Chhibber et al., 2013; Yilmaz et al., 2013). However, the effect of staggering the administration of these therapeutic agents on *S. aureus* biofilms has not been investigated.

The main aim of the current study is to investigate the ability of phage to enhance antibiotic activity against biofilm-forming *S. aureus*. Furthermore, the study aimed to elucidate whether the order in which treatment was administered had an impact on biofilm eradication outcomes.

## Materials and Methods

### Bacterial Strain and Phage

The *S. aureus* biofilm-forming strain ATCC 35556 and the lytic phage SATA-8505 were obtained from the American Type Culture Collection (ATCC). This *S. aureus* isolate served as the host strain for phage propagation. All bacterial cultures were incubated at 37°C unless otherwise stated.

### Antibiotics

Five antibiotics clinically used to treat *S. aureus* infections were assessed. These antibiotics were divided into two groups based on their mode of action. The first group consisted of vancomycin, dicloxacillin sodium salt, and cefazolin sodium salt which inhibit bacterial cell wall synthesis, while the second group consisted of linezolid and tetracycline hydrochloride which inhibit protein synthesis. All antibiotics tested in this study were obtained from Sigma–Aldrich (Canada). Stock solutions of the antibiotics were prepared in sterile double distilled water to a final concentration of 10 mg/mL, with the exception of linezolid which was prepared in dimethyl sulfoxide (Sigma–Aldrich) according to manufacturers’ recommendations. Working stocks of these antibiotics were prepared in Mueller Hinton II cation-adjusted (MH-CA).

### Minimal Inhibitory Concentration (MIC)

Minimal inhibitory concentration values were determined according to the Clinical Laboratory Standards Institute [CLSI] (2015) M107-A10 guidelines. Briefly, overnight liquid cultures of *S. aureus* were adjusted to OD_600_ = 0.1 in MH-CA media (BD Biosciences, Sparks Glencoe, MD, United States), and was further diluted in a 1:1 ratio in MH-CA. Fifty microliters of the adjusted bacterial culture was added to the wells of a 96-well plate (tissue culture treated; Falcon, Corning Inc., Durham, NC, United states) (approximately 10^5^ CFU/well). A twofold serial dilution of the antibiotics was prepared from stock solutions in MH-CA to obtain concentrations of 1024–0.125 μg/mL. The antibiotics were then added to the bacterial cell suspension (50 μL/ well) to obtain an antibiotic concentration gradient across the plate. The plates were statically incubated for 24 h at 37°C. Visible growth was monitored and MIC values were assigned.

### Establishing Biofilms

Overnight liquid bacterial cultures were adjusted to OD_600_ = 0.1 (10^7^ CFU/mL) in tryptic soy broth media supplemented with 0.5% glucose (TSBg). A 500 μL aliquot of the bacterial suspension was added to each well of 48-well polystyrene tissue culture plates (Falcon, Corning Inc., Durham, NC, United States) and incubated at 37°C statically for 24 h to allow for the formation of mature biofilms.

### Minimal Biofilm Eradication Concentration (MBEC)

Minimal Biofilm Eradication Concentration values were determined following the method described by Cui et al. (2016) with some modifications. Briefly, following mature biofilm formation (described above), planktonic cells were aspirated and 500 μL of increasing concentrations of antibiotics (32–1024 μg/mL) prepared in MH-CA was added to the biofilms and allowed to incubate at 37°C, for 24 h statically. The planktonic cells were aspirated, and the residual biofilm cells were mechanically dislodged into phosphate-buffered saline (pH 7.4), and homogenized by vigorous pipetting. The bacterial suspension was serially diluted, plated on tryptic soy broth agar (TSA), and incubated overnight at 37°C. Following incubation MBEC values were assigned. MBEC values were determined using the 48-well format since this format was used to assess antibiotic and phage interactions against biofilms.

### Assessment of Antibiotic and Phage Interactions

This assay was performed to evaluate the nature of the interactions between the tested antibiotics and the SATA-8505 phage against pre-formed biofilms of *S. aureus* ATCC 35556. The concentration of phage used for all the experiments was 10^6^ plaque forming unit (PFU)/mL [it was determined that a concentration of 10^5^ PFU/well in a total volume of 500 μL was sufficient for successful infection in this assay format (data not shown)]. The antibiotics were tested at six concentrations: 1024, 512, 256, 128, 64, and 32 μg/mL. The highest concentration of tetracycline used was 256 μg/mL, and this was due to difficulties associated with accurately quantifying viable biofilm cells caused by the bacteriostatic nature of the antibiotic. All treatments were prepared in MH-CA. Biofilms treated with MH-CA alone served as the cell control. The experiments were repeated at least three independent times.

**FIGURE 1:**
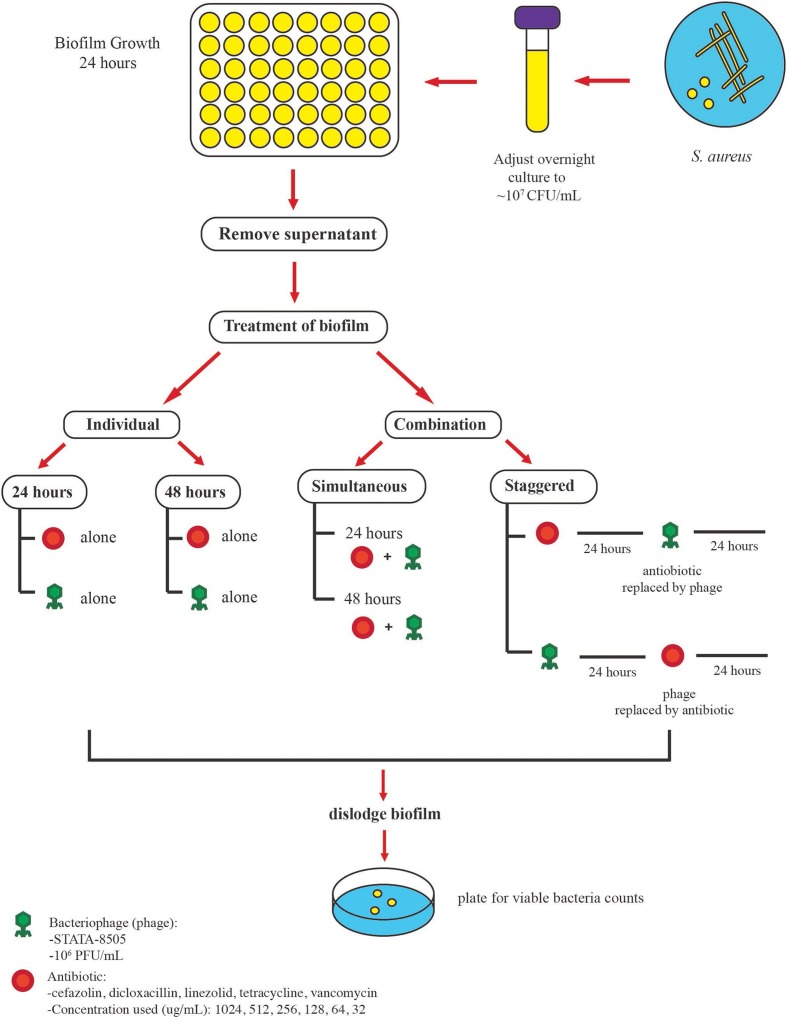
Protocol used to examine the antimicrobial activities of phage SATA-8505 and five different antibiotics against *S. aureus* ATCC 35556 biofilms. Mature biofilms were obtained after 24 h of incubation, planktonic cells were removed, and the biofilms were exposed to different treatments. The biofilm was exposed to either (a) individual treatment (phage or antibiotic alone) for 24 or 48 h or (b) a combination of phage and antibiotics. Two approaches were employed for the combination treatment, the first was to expose the biofilm to antibiotics and phage simultaneously (24 or 48 h) and the second was to stagger the exposure to phage and antibiotics over 48 h. Phage was used at a concentration of 10^6^ PFU/mL and different antibiotic concentrations were used.

Following the formation of mature biofilms the supernatant was aspirated to remove planktonic cells and the biofilm cells were treated with 500 μL of either the “individual” or “combination (simultaneous or staggered)” treatments (**Figure 1**).

In the individual format, biofilms were treated with either the phage alone (∼10^5^ PFU/well) or with the concentration gradient (described above) of antibiotics alone and incubated at 37°C for 24 or 48 h statically (**Figure 1**). Following the treatment regimen, the supernatant was removed and the biofilms were mechanically dislodged and homogenized by vigorous pipetting. The residual viable biofilm cells were enumerated by plating 10-fold serial dilutions of the bacterial cell suspensions on TSA.

In the simultaneous combined treatment format, the pre-formed biofilms were treated with 500 μL/well of the concentration gradient of antibiotics described above with the exception that the suspension contained a total phage content of ∼10^5^ PFU/500 μL. The plates were then allowed to incubate at 37°C for either 24 or 48 h.

In the staggered format, the pre-formed biofilms were first exposed to one of the treatments (phage alone or antibiotic alone) at the concentrations described above and allowed to incubate at 37°C for 24 h after which the supernatant was removed and the biofilm was then exposed to the second treatment for an additional 24 h at 37°C. The residual viable biofilm cells were enumerated as described previously.

### Statistical Analysis

Bacterial CFU counts were log_10_ transformed. Mixed model analysis was performed to determine the nature of the interaction (synergistic, additive, or antagonistic) of the combined treatment of phage and antibiotics against *S. aureus* biofilms. A *p*-value of <0.05 was considered significant. Reduction in viable bacteria counts was calculated as the difference between viable counts of the untreated biofilms (control) and the treated biofilms. An interaction was defined as being synergistic when the combination of the treatments led to greater reduction of bacteria than the sum of the individual effects. An interaction was defined as being additive in nature if the combination of the treatments resulted in bacterial reductions equal to the sum of the individual effects. While an antagonistic interaction was one that resulted in bacterial reductions that were lower than the sum of the individual effects. This could be described by the following equations:

Coefficient of interaction is equal to log (AB^R^) - ((log (A^R^) + (log (B^R^)),

where

A^R^, reduction in bacteria counts treatment A;
B^R^, reduction in bacteria counts treatment B;
AB^R^, reduction in bacterial counts following the combined treatment (AB) (staggered or simultaneous).

If the coefficient is:

$$\begin{array}{r} =\;0,\;\mathrm{additive interaction, (1)}\\ >\;0,\;\mathrm{Synergistic interaction, (2)}\\ <\;0,\;\mathrm{Antagonistic interaction. (3)} \end{array}$$

From the mixed model analysis, if the interaction was significant (*p*-value < 0.05), with a positive coefficient, it was concluded that combining the treatments resulted in a synergistic interaction (Equation 2). However, a significant interaction with a negative coefficient (Equation 3) was an indicator of an antagonistic effect. If the interaction was not significant (*p*-value > 0.05), then the combined treatment was considered to act in an additive (independent) manner against the biofilm (Equation 1).

To evaluate the possible effect of antibiotic concentrations on the efficiency of the treatment, the antibiotic concentrations were log_2_ transformed and linear regression analysis was performed. Data analyses were performed with computer software [Statistical Analysis System (SAS) 2002–2010, SAS Institute, Inc., Cary, NC, United States].

## Results

### Anti-biofilm Activity of the Simultaneous Treatment of Antibiotics and Phage

Pre-formed biofilms were simultaneously treated with antibiotics and SATA 8505 over 24 and 48 h. The MIC and MBEC values of the five tested antibiotics were determined (**Table 1**), and it was found that the MBEC of all the antibiotics were >1024 μg/mL.

**Table 1:** MIC and MBEC values (μg/mL) of different antibiotics against *S. aureus* ATCC 35556.

Vancomycin		Cefazolin		Tetracycline		Linezolid		Dicloxacillin	
MIC	MBEC	MIC	MBEC	MIC	MBEC	MIC	MBEC	MIC	MBEC
4	>1024	0.5	>1024	0.5	>1024	4	>1024	0.125	>1024

The antibiotic concentrations used in the successive experiments which evaluated antibiotic and phage interactions were the same range utilized for MBEC determination. Following the 24 h incubation period, the combination treatment of SATA-8505 with linezolid or tetracycline demonstrated an additive effect at all concentration of antibiotics tested (*p* > 0.05, **Table 2**). Vancomycin showed a similar pattern at lower concentrations (*p* > 0.05), however at concentrations higher than 64 μg/mL, the interaction with the phage was mostly antagonistic in nature (*p* < 0.05, **Table 2**). An antagonistic interaction was observed at all concentrations of cefazolin and dicloxacillin tested as well (*p* < 0.05, **Table 2**).

**Table 2:** Interactions between SATA-8505 and different antibiotics exhibited when *S. aureus* biofilms were exposed to the simultaneous treatment of these two agents over 24 h.

Concentration (μg/mL)		Antibiotic				
		Vancomycin	Dicloxacillin	Cefazolin	Tetracycline	Linezolid
		**Interaction**				
32		A	G	G	A	A
64		A	G	G	A	A
128		G	G	G	A	A
258		G	G	G	A	A
512		G	G	G	n/a	A
1024		A	G	G	n/a	A

*A, additive interaction; G, antagonistic interaction; n/a, not applicable*.

**FIGURE 2:**
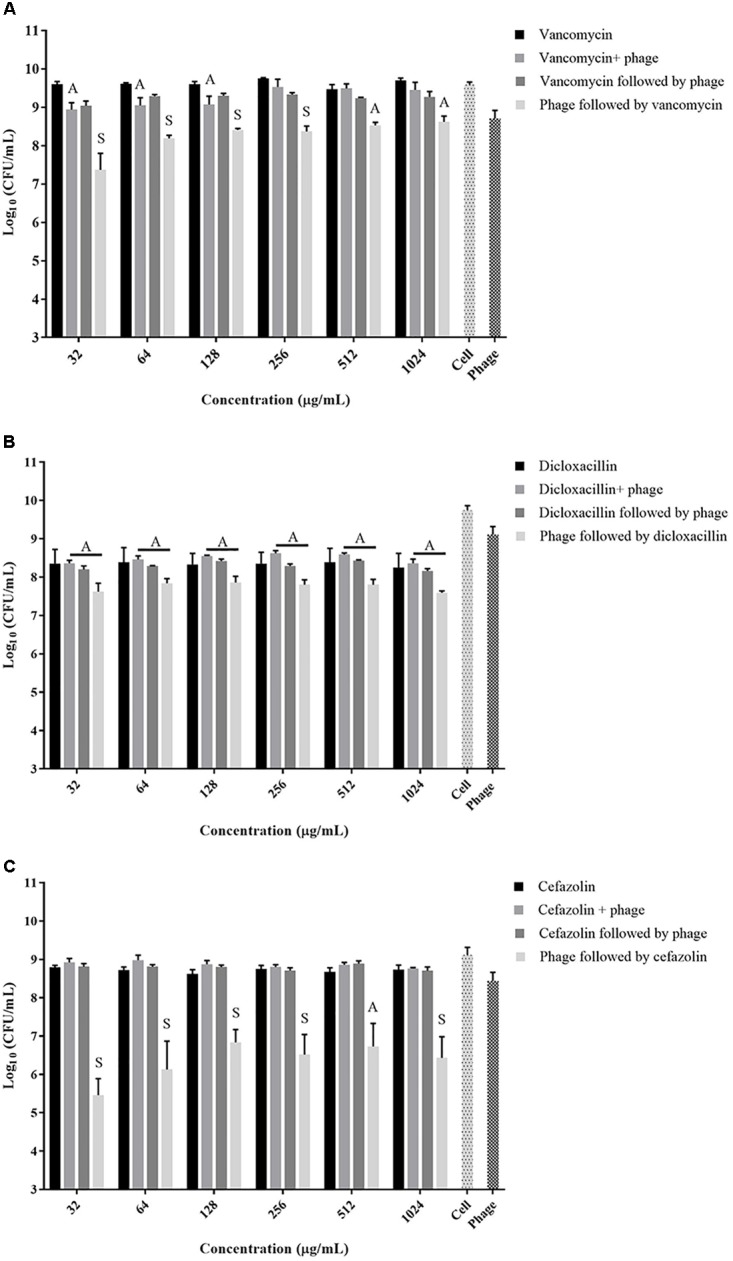
Viable *S. aureus* ATCC 35556 counts following 48 h exposure to phage SATA-8505 and different concentrations of the cell wall synthesis inhibitor antibiotics; **(A)** vancomycin, **(B)** dicloxacillin, or **(C)** cefazolin. The treatment strategies employed were: antibiotic alone, phage alone, phage and antibiotic simultaneously, phage first followed by antibiotic, or antibiotic first followed by phage. Synergistic (S) and additive (A) interactions have been indicated. Bacterial cells treated with Mueller Hinton cation-adjusted (MH-CA) alone served as a control (cell). *N* = 3–4 ±*SD*.

**FIGURE 3:**
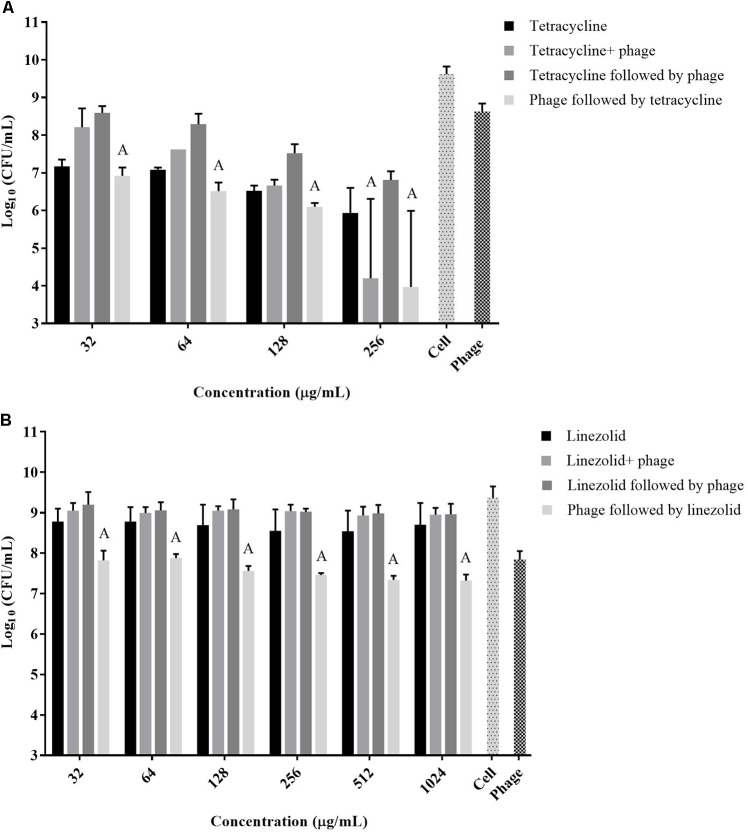
Viable *S. aureus* ATCC 35556 counts 48 h following exposure to phage SATA-8505 and different concentrations of antibiotics that inhibit translation; **(A)** tetracycline and **(B)** linezolid. The treatment scenarios: antibiotic alone, phage alone, phage and antibiotic simultaneously, phage first then followed by antibiotic, or antibiotic first followed by phage. Synergistic (S) and additive (A) interactions have been indicated. Bacterial cells treated with MH-CA alone served as a control (cell). *N* = 3 ±*SD*.

The exposure of biofilms to cefazolin, linezolid, or tetracycline in combination with the phage over 48 h resulted mostly in an antagonistic effect (**Figures 2C**, **3A,B**) (*p* < 0.05) with no major reductions of bacterial viable counts being observed. In the case of vancomycin, an additive interaction was observed when the antibiotic was used in conjunction with the phage up to a concentration of 128 μg/mL (*p* > 0.05). At concentrations of 256 μg/mL and higher, an antagonistic interaction was observed (**Figure 2A**) (*p* < 0.05). Of the antibiotics tested, dicloxacillin alone exhibited additive interactions at all concentrations tested (**Figure 2B**) (*p* > 0.05). Therefore, results indicate that simultaneous phage and antibiotic treatments for either 24 or 48 h have limited antibacterial activity against *S. aureus* biofilms.

### Staggered Treatment of the Phage and the Antibiotic

A strategy to stagger the phage and antibiotic treatments against biofilms was employed to assess the effect that the order of treatment may have on biofilm viability. Exposing pre-formed biofilms to antibiotics (vancomycin, cefazolin, tetracycline, or linezolid) prior to phage treatment resulted in antagonistic interactions between the two agents at all concentrations tested (**Figures 2A,C**, **3A,B**) (*p* < 0.05). On the other hand, when the phage treatment preceded exposure to either vancomycin or cefazolin, significant anti-biofilm activities were observed corresponding to a synergistic interaction between the antibiotics and the phage (**Figures 2A,C**) (*p* < 0.05). The exposure of biofilms to phage prior to either linezolid or tetracycline gave rise to levels of biofilm reduction indicative of an additive interaction (**Figures 3A,B**) (*p* > 0.05). The data suggest that biofilm exposure to phage prior to antibiotics is more effective at eliminating biofilm-associated cells. Interestingly, dicloxacillin and the phage interacted in an additive manner regardless of whether they were exposed to the biofilm simultaneously or in a staggered fashion (**Figure 2B**) (*p* > 0.05).

### Antibiotic Concentration and Staggered Treatment Efficiency

**FIGURE 4:**
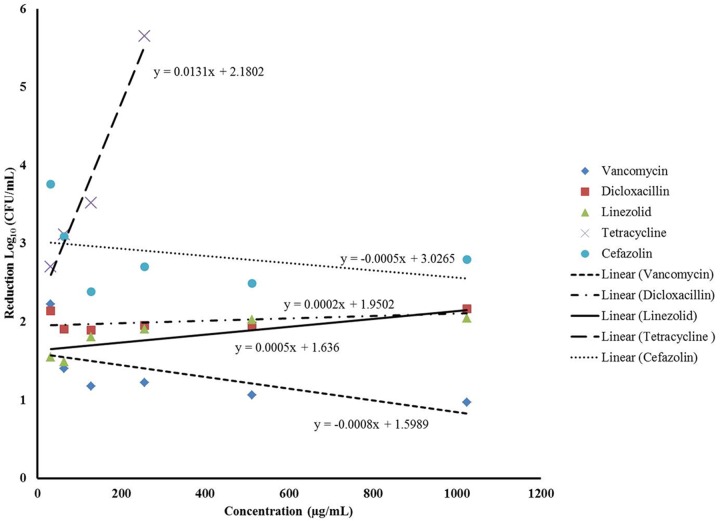
Linear regression analysis assessing the correlation between the different concentrations of the antibiotics and the average biofilm reduction observed during the staggered treatment where the phage preceded antibiotics.

Since our data indicated that better biofilm reduction outcomes can be achieved when phage is administered prior to antibiotics, we investigated the effect of the antibiotic concentrations on enhancing the staggered treatment efficiency. Linear regression analysis of the data demonstrated that there was linear relationship between the concentration of most antibiotics and biofilm reduction (**Figure 4**). The biofilm reduction observed was directly proportional to the concentration of linezolid and tetracycline used (*p* = 0.0019). However, the biofilm reduction observed for vancomycin was enhanced when lower concentrations were used (*p* = 0.0014). In the case of cefazolin, an inversely proportional relationship between antibiotic concentration and anti-biofilm effect was observed up to a concentration of 128 μg/mL. No linear correlation was observed between the concentration of dicloxacillin and tetracycline employed and biofilm elimination (*p* = 0.6791 and *p* = 0.0654, respectively).

## Discussion

The emergence of multi-drug-resistant bacteria has been on the rise over the past decade; a problem that has been compounded by a significant decline in the discovery of novel antibiotics. Consequently, there has been a resurgent interest in harnessing the antimicrobial properties of phages as a therapeutic platform.

*Staphylococcus aureus* biofilms are the leading cause of clinical infections such as osteomyelitis and infections associated with artificial implants (Archer et al., 2011; Tande and Patel, 2014). The successful treatment of biofilm-related infections using current antibiotic therapy continues to be a major challenge. Therefore, alternative therapeutic platforms that utilize phage to overcome the biofilm state offer a promising alternative to traditional antibiotic treatments (Cornelissen et al., 2011; Gutiérrez et al., 2012).

Different groups have investigated the effectiveness of combining antibiotics with phage to eradicate bacterial populations existing in both planktonic and biofilm states. Zhang and Buckling (2012) demonstrated that when planktonic cultures of *Pseudomonas fluorescens* had been exposed to a combination of lytic phage and antibiotics, it resulted in a higher reduction in viable cells when compared with cultures treated with antibiotics alone. The simultaneous addition of phage to antibiotic regimens has also been reported to have a fascinating outcome of enhancing the sensitivity of multi-drug-resistant bacteria to antibiotics (Chan et al., 2016). Studies have also assessed how planktonic cultures are affected when these agents are added sequentially. Of note, groups led by Escobar-Paramo and Torres-Barcelo reported that the order in which the treatment was administered impacted bacterial reduction outcomes and antibiotic resistance profiles in the planktonic populations of *Pseudomonas* spp. studied (Escobar-Páramo et al., 2012; Torres-Barceló et al., 2014). Torres-Barceló et al. (2014) also claimed that the order in which the treatment was administered affected resistance profiles to a greater extent than the antibiotic dose employed.

According to the National Institute of Health, up to 80% of chronic infections are biofilm related (National Institute of Health [NIH], 2007). Consequently, strategies aimed at eradicating biofilms are of clinical significance. A majority of the studies that have investigated the effects of antibiotic and phage treatment on biofilm eradication have administered the two antibacterial agents simultaneously (Bedi et al., 2009; Verma et al., 2009, 2010). These studies have demonstrated that the efficacy of such treatments vary, and is dependent on the antibiotic, bacteria, and the phage employed. However, the value of utilizing phage to augment antibiotic effects cannot be underestimated. Bedi et al. (2009) demonstrated that a significant reduction in biofilm mass could be achieved *in vitro* following combined therapy as a result of phage-mediated biofilm disruption. Additionally, successful *in vivo* studies and clinical data in human subjects highlight the promise of using phage in conjunction with antibiotics to treat recalcitrant infections such as *Staphylococcus* sepsis, lung infection, and osteomyelitis (Chhibber et al., 2013; Yilmaz et al., 2013; Kaur et al., 2016). A key study by Chaudhry et al. (2017) investigated different strategies of administrating phage and antibiotics to treat *P. aeruginosa* biofilms. They were able to demonstrate that the order in which the two treatments were administered greatly affected biofilm eradication outcomes (Chaudhry et al., 2017). This, to our knowledge, is the only study that has investigated the effect that an order of treatment has on biofilm eradication.

**FIGURE 5:**
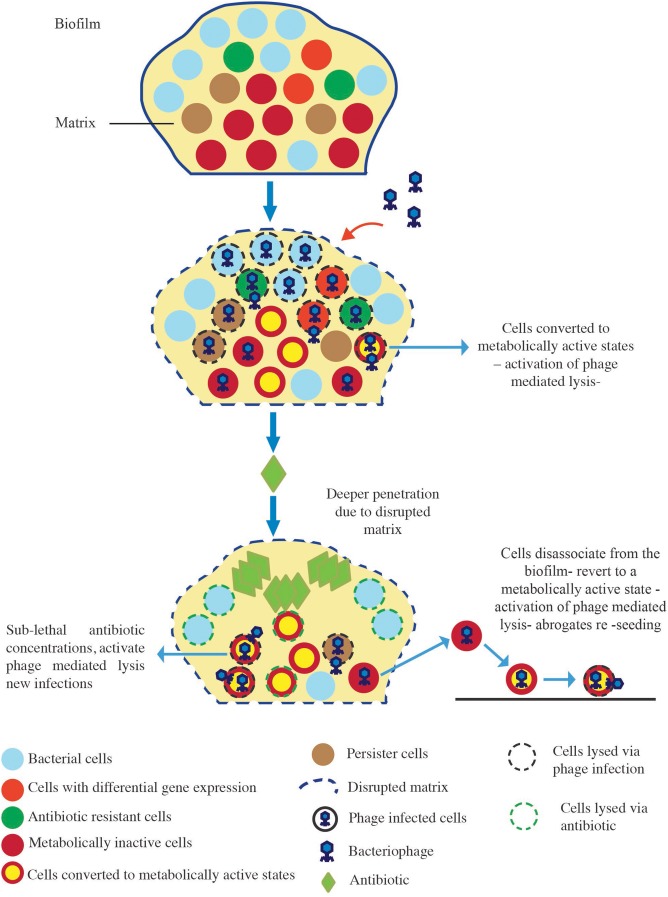
Proposed model of biofilm reduction by the “phage first” staggered treatment. Exposure of the biofilm to phage first results in the disruption of the matrix, and the phage mediated lysis of biofilm-associated cells including antibiotic-resistant cells, differentially regulated cells, metabolically inactive cells, and persister cells. The addition of antibiotics to such a system leads to enhanced antibacterial effects due to higher local concentrations resulting from deeper penetration of these agents. Sub-lethal concentrations experienced by phage-infected cells in deeper layers of the biofilm elicit the activation of phage-mediated lysis resulting in further bacterial reductions.

In the current study, we assessed whether phage can augment the activity of five antibiotics against *S. aureus* biofilms *in vitro*. In addition, the effectiveness of sequential or simultaneous administration of the treatments was compared. Our data demonstrated that the order in which *S. aureus* biofilms were exposed to the treatments was a key determinant of biofilm reduction outcomes. An analysis of the interplay between the antibiotics and the phage during simultaneous treatment demonstrated that of the five antibiotics tested, only dicloxacillin displayed additive interactions with the phage, while the other four antibiotics displayed predominantly antagonistic interactions at the different concentrations tested. This suggested that the interaction between phage and antibiotics was dependent on the antibiotic being studied. This observation is in line with other *in vitro* studies that showed varying degrees of biofilm reduction when biofilms were treated simultaneously with antibiotics and phage (Verma et al., 2009, 2010; Chaudhry et al., 2017). Our findings gave credence to investigating other strategies that could be used to bolster the effects of phage and antibiotics. Consequently, we demonstrated that establishing a phage infection in the biofilm prior to antibiotic exposure led to the highest *S. aureus* biofilm reductions. Our results also highlighted that biofilm reduction after phage treatment was dependent on the type and concentration of antibiotics utilized. Furthermore, we were able to demonstrate that this strategy paved the way for synergistic interactions to occur between the phage and two antibiotics (vancomycin and cefazolin), leading to the highest biofilm reductions observed. Our results were comparable to those reported by Chaudhry et al. (2017) who observed enhanced eradication of *P. aeruginosa* biofilms that were pre-exposed to phage prior to gentamycin or tobramycin. Their group was also able to recover high phage titers when biofilms were treated with phage prior to antibiotic exposure, which translated to enhanced anti-biofilm activity. However, much lower phage titers were obtained when other treatment strategies were employed, and were accompanied with lower anti-biofilm efficiencies. The treatment of biofilms with phage prior to antibiotics allows phages to rapidly replicate in the bacterially dense environment of the biofilm leading to high phage densities and the disruption of the biofilm matrix (Chaudhry et al., 2017). The addition of antibiotics to such a system leads to more robust bacterial reduction owing to the deeper penetration of these agents. However, when biofilms are exposed to antibiotics first followed by phage, the bacterial populations available for phage infection are reduced which can negatively affect infection kinetics and ultimately affect eradication outcomes (Payne and Jansen, 2000; Escobar-Páramo et al., 2012). Taken together, these factors can account for the heightened anti-biofilm activity observed in our study when a sequential (phage first) treatment approach was employed (**Figure 5**). Further investigation is needed to determine the precise mechanisms involved in the observed biofilm reduction.

The mode of action of the antibiotics being used in conjunction with phage may have contributed to the outcomes observed in our study. Cefazolin and vancomycin are both cell wall synthesis inhibitors, and when they were administered to biofilms following phage treatment it resulted in markedly higher biofilm eradication outcomes, especially at the lower concentrations tested. This can be explained by the fact that sub-lethal concentrations of antibiotics that affect cell wall integrity have been reported to activate the bacterial stress response resulting in an up-regulation of phage replication and cell lysis (Comeau et al., 2007; Chaudhry et al., 2017). The reduction in anti-biofilm activity observed at higher concentrations can be caused by diminished bacterial densities which curtail new phage infections, thereby diminishing the overall anti-biofilm effect. Interestingly, although dicloxacillin also affects cell wall integrity, a robust anti-biofilm reduction was not achieved when used in conjunction with phage. This observation suggested that other factors may play a role in biofilm elimination in combination therapy.

In this study, we have demonstrated that the antibiotic-mediated eradication of *S. aureus* biofilms can be augmented if a bacteriophage infection is established prior to antibiotic treatment. These results are the first to document the impact that an order of treatment has on *S. aureus* biofilm eradication. Our study shed light on the importance of investigating the effect treatment order can have in optimizing phage–antibiotic treatment efficacy against biofilms. Furthermore, results from the current study suggested that the success of such a treatment regimen depends on numerous factors including: the nature of the interaction between the phage and antibiotic, the type of antibiotic, and the concentration of antibiotic employed. Our findings provide a basis for parameters to be considered while assessing phage antibiotic pairings for the treatment of biofilm-related infections. Our work, as well as other *in vitro* studies, highlights the potential clinical benefit of combination therapies using libraries of lytic phages and antibiotics to treat biofilm-related infections.

## Author Contributions

DK and MT designed and conducted the experiments, analyzed the data, and drafted the manuscript. HA, QY, SR-A, J-SD, and AC contributed to the conception and design of the work and revised the manuscript critically.

## Conflict of Interest Statement

The authors declare that the research was conducted in the absence of any commercial or financial relationships that could be construed as a potential conflict of interest.
